# FOCUS: object-centric world models for robotic manipulation

**DOI:** 10.3389/fnbot.2025.1585386

**Published:** 2025-04-30

**Authors:** Stefano Ferraro, Pietro Mazzaglia, Tim Verbelen, Bart Dhoedt

**Affiliations:** ^1^IDLab, Department of Information Technology, Ghent University–imec, Ghent, Belgium; ^2^VERSES Research Lab, VERSES, Los Angeles, CA, United States

**Keywords:** world models, object-centric representation, neuro robotics, object-centric exploration, embodied-AI

## Abstract

Understanding the world in terms of objects and the possible interactions with them is an important cognitive ability. However, current world models adopted in reinforcement learning typically lack this structure and represent the world state in a global latent vector. To address this, we propose FOCUS, a model-based agent that learns an object-centric world model. This novel representation also enables the design of an object-centric exploration mechanism, which encourages the agent to interact with objects and discover useful interactions. We benchmark FOCUS in several robotic manipulation settings, where we found that our method can be used to improve manipulation skills. The object-centric world model leads to more accurate predictions of the objects in the scene and it enables more efficient learning. The object-centric exploration strategy fosters interactions with the objects in the environment, such as reaching, moving, and rotating them, and it allows fast adaptation of the agent to sparse reward reinforcement learning tasks. Using a Franka Emika robot arm, we also showcase how FOCUS proves useful in real-world applications. Website: focus-manipulation.github.io.

## 1 Introduction

In our daily lives, we effortlessly interact with objects to accomplish a wide range of tasks. Through these interactions, we instinctively infer an object's identity, spatial position, 3D structure, appearance, and texture, effectively building a generative model of how objects are formed (Parr et al., 2021). For robot manipulators, replicating these tasks presents a significant challenge due to the intricate and dynamic nature of interactions between the agent and its environment.

In recent years, deep reinforcement learning (RL) has shown to be a promising approach for dealing with complex manipulation scenarios (Levine et al., 2016; OpenAI et al., 2019; Kalashnikov et al., 2018; Lu et al., 2021; Lee et al., 2021; Ferraro et al., 2022a). Among RL algorithms, model-based approaches aspire to provide greater data efficiency, compared to the model-free counterparts (Fujimoto et al., 2018; Haarnoja et al., 2018). Adopting world models (Ha and Schmidhuber, 2018; Hafner et al., 2021), i.e. generative models that learn the environment's dynamics by reconstructing sensory observations, model-based agents have shown impressive performance across several domains (Hafner et al., 2021; Rajeswar et al., 2023; Hafner et al., 2023), including real-world applications, such as robotic manipulation and locomotion (Wu et al., 2022). However, world models that indistinctly reconstruct all information in the environment can suffer from several failure modes. For instance, in visual tasks, they can ignore small, but important features for predicting the future, such as small objects (Seo et al., 2022). They also tend to waste the model capacity on visually rich, but irrelevant features, such as static backgrounds (Deng et al., 2022). In the case of robotic manipulation, this is problematic because the agent strongly needs to acquire information about the objects to manipulate to solve a given task.

Another challenge in RL for manipulation is engineering reward functions that drive the agent's learning toward task completion. Attempting to design dense reward functions easily leads to faulty reward designs (Amodei et al., 2016; Clark and Amodei, 2016; Krakovna et al., 2020; Popov et al., 2017). One solution is to adopt sparse reward feedback, providing a positive reward only for successful task completion. However, these functions are challenging to optimize with RL, due to the difficulty of finding such rewards in the environment. Thus, they require appropriate exploration strategies, for which previous work has resorted to artificial curiosity mechanisms (Oudeyer et al., 2007; Schmidhuber, 1991) or entropy maximization strategies (Mutti et al., 2021; Liu and Abbeel, 2021). In Liu and Abbeel (2021), exploration emerges by maximizing the entropy over the full latent representation, resulting in the agent potentially focusing on exploring irrelevant aspects of the scene (Burda et al., 2018b).

Humans, on the other hand, tend to develop a structured mental model of the world by interacting with objects registering specific features associated with objects, such as shape, color, etc. (Hawkins et al., 2017; Ferraro et al., 2023). Since infancy, toddlers learn this by actively engaging with objects and manipulating them with their hands, discovering object-centric views that allow them to build an accurate mental model (Smith et al., 2018; Slone et al., 2019; Ferraro et al., 2022b).

In this work, we present an approach inspired by the principle that objects should be of primary importance in an agent's world model, and motivated by the above issues regarding: i) the complexity of modeling object entities in the environment, and ii) the necessity of autonomously discovering interactions with such objects. We introduce **FOCUS**, a model-based RL agent that learns an object-centric representation of the world. Unlike holistic scene representations, an object's latent vector allows the agent to prioritize information about objects. Leveraging the object-centric representation, it's possible to design an exploration strategy that focuses on the interactions where objects are involved. Crucially, the proposed focused exploration strategy allows for improved performance on sparsely rewarded tasks when compared to the state-of-the-art.

Our contributions in this work can be summarized as:

- an object-centric world model, which learns the latent dynamics of the environment where the information about objects is discriminated into distinct latent vectors;
- an object-centric exploration strategy, which encourages interactions with the objects, by maximizing the entropy of the latent object's representation;
- empirical evaluation of the approach, showing how object-centric models improve the agent's understanding of the objects in the scene and how the object-centric exploration strategy fosters interaction with the objects. This leads the agent to more efficiently solve robotic manipulation tasks in several settings and tasks, including ManiSkill2 (Gu et al., 2023), robosuite (Zhu et al., 2020) and Metaworld (Yu et al., 2019) environments.
- a deployment on a real robotic platform, showcasing the possibility of successfully applying our approach to a hardware-based setup.

## 2 Background

### 2.1 Reinforcement learning and world models

In RL, the agent receives inputs *x* from the environment and can interact through actions *a*. The objective of the agent is to maximize the discounted sum of rewards $\underset{t}{\sum }\gamma^{t}r_{t}$, where *t* indicates discrete timesteps. In order to do so, RL agents learn an optimal policy π(*a*|*x*) outputting actions that maximize the expected cumulative discounted reward over time, generally estimated using a critic function, which can be either a state-value function or an action-value function (Haarnoja et al., 2018; Fujimoto et al., 2018). In addition, model-based RL methods learn a model of the transition dynamics of the environment and use it to select actions (Hansen et al., 2022) or to optimize the actor-critic networks (Janner et al., 2021). Recently, world models (Ha and Schmidhuber, 2018) have adopted deep generative models (Goodfellow et al., 2016) to learn the dynamics of the environment, capturing the environment dynamics into a latent space, which can be used to learn the actor and critic functions using imaginary rollouts (Hafner et al., 2021, 2023) or to actively plan at each action (Schrittwieser et al., 2020; Rajeswar et al., 2023; Song et al., 2024). Given that world model-based RL has been shown to be more efficient than model-free RL (Hafner et al., 2023) and the importance of sample-efficiency in robotic manipulation, we base our work on world models and RL for learning behaviors from interactions.

### 2.2 Exploration

Solving sparse-reward tasks is a hard problem in RL because of the difficulty of exploring the environment and identifying rewarding states. Inspired by artificial curiosity theories (Schmidhuber, 1991; Oudeyer et al., 2007), several works have designed exploration strategies for RL (Pathak et al., 2017; Mazzaglia et al., 2022; Rajeswar et al., 2021). Other exploration strategies that have shown great success are based upon the ideas of maximizing uncertainty (Pathak et al., 2019; Sekar et al., 2020), or the entropy of the agent's state representation (Liu and Abbeel, 2021; Seo et al., 2021; Mutti et al., 2021). One issue with exploration in visual environments is that these approaches can be particularly attracted by easy-to-reach states that strongly change the visual appearance of the environment (Burda et al., 2018a). In robotic manipulation, this can cause undesirable behaviors, e.g., a robot arm exploring different poses in the proximity of the camera but ignoring interactions with the objects in the workspace (Rajeswar et al., 2023). Our method, instead, leverages object-centric representations to encourage agents to interact with the objects present in the scene. By designing an object-centric exploration strategy, we provide a better alternative to curiosity mechanisms for robotic manipulation, which have no specific targets for exploration in the environment.

### 2.3 Object-centric representations

Decomposing scenes into objects can enable efficient reasoning over high-level building blocks and ensure the agent focuses on the most relevant concepts (Dittadi et al., 2021). Several 2D object-centric representations, based on the principle of representing objects as separate entities within the model, have recently emerged (Locatello et al., 2020; Greff et al., 2020; Burgess et al., 2019; Nakano et al., 2023). Due to computational and quality constraints, these object-centric representations have not been extended to more complex scenarios, where the interaction with an agent is also to be modeled. Related work investigated the usefulness of object-centric representations for control, using model-free RL (Diuk et al., 2008; Janner et al., 2019; Kipf et al., 2020; Yoon et al., 2023). Inspired by these approaches, we propose an object-centric world model which allows us to learn behaviors efficiently by leveraging model-based RL. The object-centric representation improves the agent's predictions about objects and can be used both to enable more accurate control, e.g. to solve dense-rewards RL tasks, and to foster interactions with objects using a new object-centric exploration strategy, e.g. in sparse-rewards RL tasks. The closest work in literature to our approach (Sancaktar et al., 2022) is an object-centric exploration strategy based on graph-structured models for control. However, this approach requires already precise information about objects, e.g. the position, which is generally available only in simulation. Instead, our approach is designed to work well for common visual manipulation settings, where information about the scene is provided to the agent only through camera images.

## 3 Object-centric world model

The agent observes the environment through the inputs *x*_*t*_ = {*o*_*t*_, *q*_*t*_} it receives at each interaction, where we can distinguish the (visual) observations *o*_*t*_, e.g. camera RGB, from the proprioceptive information *q*_*t*_, e.g. the robot joint states and velocities. This information is processed by the agent through an encoder model *e*_*t*_ = *f*(*x*_*t*_), which can be instantiated as the concatenation of the outputs of a CNN for high-dimensional observations and an MLP for low-dimensional proprioception.

**Figure 1:**
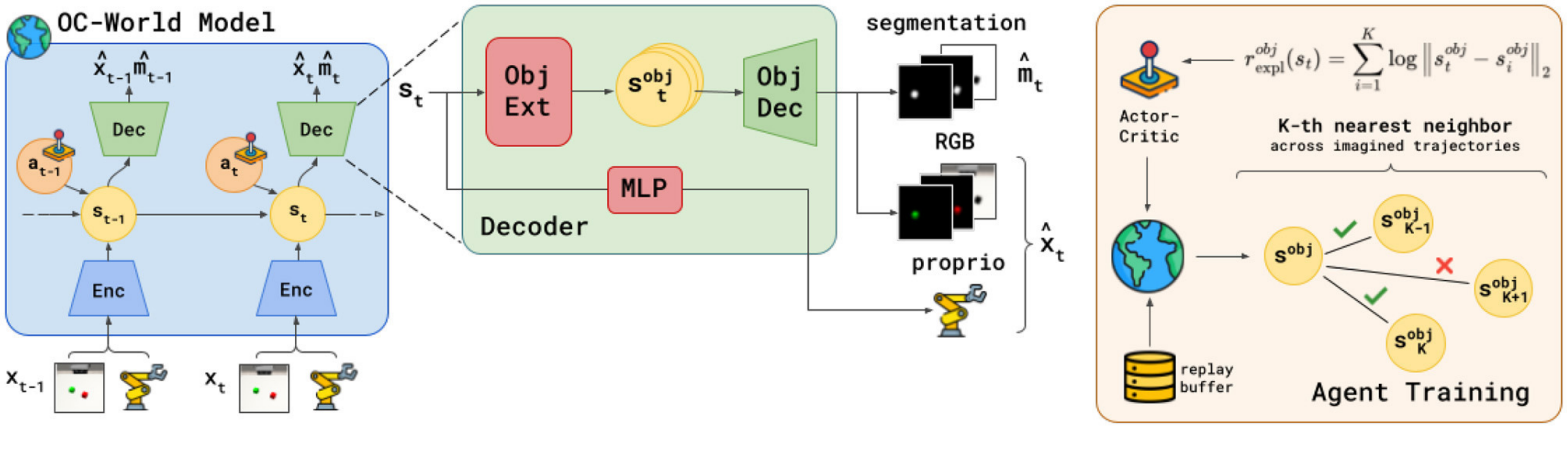
Overview of FOCUS. The agent learns a structured world model **(left)** that disentangles information in the environment by learning to reconstruct masked information about each observed object, thanks to an object-centric decoder **(center)**. The learned object-centric state representation is used to train a policy that incentivizes object-centric exploration **(right)**, maximizing the entropy of the object representation as a form of intrinsic reward.

The world model aims to capture the dynamics of the inputs into a latent state *s*_*t*_. In previous work (Hafner et al., 2021), this is achieved by reconstructing the inputs using an observation decoder. With FOCUS, we are interested in separating object-specific information into separate latent representations $s_{t}^{obj}$. For this reason, we instantiate two object-conditioned components: an *object latent extractor* and an *object decoder*. We first describe the structure and loss of the world model (in Figure 1, **left**) before delving into more details about the novel object-centric components of FOCUS (in Figure 1, **center**).

### 3.1 World model

Overall, the learned world model is composed of the following components:


$$\begin{array}{ll} \text{Encoder:} & \;e_{t}=f\left(x_{t}\right),\\ \text{Posterior:} & \;p_{\phi }\left(s_{t+1}|s_{t},a_{t},e_{t+1}\right),\\ \text{Prior:} & \;p_{\phi }\left(s_{t+1}|s_{t},a_{t}\right),\\ \text{Proprio decoder:} & \;p_{\theta }\left(\hat{q_{t}}|s_{t}\right),\\ \text{Object latent extractor:} & \;p_{\theta }\left(s_{t}^{obj}|s_{t},c^{obj}\right),\\ \text{Object decoder:} & \;p_{\theta }\left({ô}_{t}^{obj},l_{t}^{obj}|s_{t}^{obj}\right). \end{array}$$

which are trained end-to-end by minimizing the following loss:


(1) $$\begin{array}{l} L_{\text{wm}}=L_{\text{dyn}}+L_{\text{proprio}}+L_{\text{obj}}. \end{array}$$

We explain each component in details in the following paragraphs.

For the dynamics component, i.e., prior and posterior, we adopt a recurrent state-space model (RSSM) (Hafner et al., 2019), which extracts a latent state *s*_*t*_ made of a deterministic and a stochastic component. The parameters of the RSSM modules are collectively denoted as ϕ. The dynamics minimize the Kullback-Leibler (KL) divergence between posterior and prior:


(2) $$\begin{array}{l} L_{\text{dyn}}=D_{\text{KL}}\left[p_{\phi }\left(s_{t+1}|s_{t},a_{t},e_{t+1}\right)\|p_{\phi }\left(s_{t+1}|s_{t},a_{t}\right)\right]. \end{array}$$

All parameters of the decoding units of the network are represented by θ. Proprioceptive information $\hat{q_{t}}$ is decoded out of the latent state *s*_*t*_, using an MLP. The proprioceptive decoder learns to reconstruct proprio states, by minimizing a negative log-likelihood (NLL) loss:


(3) $$\begin{array}{l} \begin{array}{c} L_{\text{proprio}}=-\log p_{\theta }\left(\hat{q_{t}}|s_{t}\right) \end{array} \end{array}$$

### 3.2 Object-centric modules

The latent state of the world model tends to compress all the information from the environment in a unique latent structure. Our intention in FOCUS is to disentangle such information into separate latent structures, learning an object-centric world model.

For each object in the scene, the *object latent extractor* receives the model latent state *s*_*t*_ and a (one-hot) vector identifying the object *c*^*obj*^, and extracts an object-centric latent $s_{t}^{obj}$. Given such an object latent, the *object decoder* reconstructs object-related observation information by outputting two kinds of information: one-dimensional “object logits” $l_{t}^{obj}$, which are used to build a segmentation mask of the scene, and object-specific observation ${ô}_{t}^{obj}$, where the information that is irrelevant to the object is masked out through the segmentation. *How is the segmentation mask learned?* The object decoder outputs one-dimensional “object logits” $l_{t}^{obj}$, which represent object-specific per-pixel logits. These logits are aggregated in a scene by applying a softmax among all object weights. The overall segmentation mask is obtained as:


(4) $$\begin{array}{l} \hat{m_{t}}=\text{softmax}\left(l_{t}^{1},...,l_{t}^{N}\right) \end{array}$$

with *N* being the object instances. Object-specific masks can be obtained by taking the corresponding object's channel mask in the segmentation. Defining object-specific masks as $m_{t}^{\text{obj}}$, we can multiply the observation by these masks, to obtain object-specific observations ${ô}_{t}^{obj}$ that focus only on the *obj*-th object information. ^1^

The object decoder loss is defined as follows:


(5) $$\begin{array}{l} \begin{array}{c} L_{\text{obj}}=-\log\underset{\text{mask}}{\underset{︸}{p\left(\hat{m_{t}}\right)}}-\log\overset{N}{\underset{\text{obj}=0}{\sum }}\underset{\text{masked reconstruction}}{\underset{︸}{m_{t}^{\text{obj}}p_{\theta }\left({\hat{x}}_{t}^{\text{obj}}|s_{t}^{\text{obj}}\right)}} \end{array} \end{array}$$

By minimizing the NLL of the masked reconstruction term, the object-decoder ensures that each object latent *s*^*i*^ focuses on capturing only its relevant information, as the reconstructions obtained from the latent are masked per object. Furthermore, objects compete to occupy their correct space in the scene (in pixel space), through the *mask* loss.

*How are the segmentation mask targets for the mask loss obtained?* In order to discriminate object information into different latent vectors, the object-centric components leverage an object discrimination process that entails learning to segment the scene observations. Some simulated robotic environments make this information available, however, the same process is non-trivial in real-world settings.

The increasing availability of large pre-trained models for segmentation offers an opportunity to avoid the problem. Thus, in our experiments, we adopt an efficient implementation of the Segment Anything Model (fastSAM; Kirillov et al., 2023; Zhao et al., 2023). At the beginning of each episode, per object segmentation instances are generated with fastSAM, using box or text prompts. For subsequent frames, segmentation maps are produced by a tracking model, for which we ground on the XMem model (Yang et al., 2023). This strongly simplifies the process of obtaining segmentation masks in robotic workspaces.

## 4 Object-centric exploration

State maximum entropy approaches for RL (Mutti et al., 2021; Seo et al., 2021; Liu and Abbeel, 2021) learn an environment representation, on top of which they compute an entropy estimate that is maximized by the agent's actor to foster exploration. Given our object-centric representation, we can incentivize well-directed exploration toward object interactions and the discovery of novel object views, by having the agent maximize the entropy over the object latent state representation.

In order to estimate the entropy value over batches, we apply a K-NN particle-based estimator (Singh et al., 2003) on top of the object latent representation. By maximizing the overall entropy, with respect to all objects in the scene, we derive the following reward for object-centric exploration:


(6) $$\begin{array}{lll} r_{\text{expl}}=\overset{N}{\underset{obj=0}{\sum }}r_{\text{expl}}^{obj}\\ \text{where }r_{\text{expl}}^{obj}\left(s\right) & \propto & \overset{K}{\underset{i=1}{\sum }}\log\|s^{obj}-s_{i}^{obj}|{|}_{2} \end{array}$$

where *s*^*obj*^ is extracted from *s* using the object latent extractor, $s_{i}^{obj}$ is the *i*-th nearest neighbor to *s*^*obj*^.

Crucially, as we learn an (object-centric) world model we can use it to optimize actions by learning actor and critic in imagination (Hafner et al., 2021), so that the latent states in Equation 6 are states of imaginary trajectories, generated by the world model by following the actor's predicted actions.

Learning actor-critic in imagination allows one to efficiently learn actions by generating hypothetical trajectories in the agent's latent state space. This can be done by applying RL for learning an actor policy π(*a*_*t*_|*s*_*t*_) that outputs actions that maximize the following bootstrapped λ-returns (Hafner et al., 2023):


(7) $$\begin{array}{l} R_{t}^{\lambda }=r_{t}+\gamma \left(\left(1-\lambda \right)v\left(s_{t+1}\right)+\lambda R_{t+1}^{\lambda }\right) \end{array}$$

with the value function *v*(*s*_*t*_) learning to approximate $R_{t}^{\lambda }$.

Given the above, we can learn an exploration actor-critic:


(8) $$\begin{array}{l} \begin{array}{c} \text{Exploration actor: }\pi_{\text{expl}}\left(a_{t}|s_{t}\right), \end{array}\;\begin{array}{c} \text{Exploration critic: }v_{\text{expl}}\left(s_{t}\right), \end{array} \end{array}$$

that learns to maximize the exploration reward in Equation 6.

FOCUS acts at two levels: it explores to find useful interactions, and consequently it learns to perform downstream tasks using the sparse rewards found in the environment.

Indeed, as the agent explores the environment, it may encounter important information that may be a source of (sparse) reward, (e.g. opening a drawer). To exploit such information, while we keep exploring, we concurrently train a *task* reward predictor *r*_task_(*s*_*t*_) and actor-critic, which can be used for solving the pre-defined task after exploring the environment, in a zero-shot or few-shot fashion.

The task actor-critic is defined as follows:


(9) $$\begin{array}{l} \begin{array}{c} \text{Task actor: }\pi_{\text{task}}\left(a_{t}|s_{t}\right), \end{array}\;\begin{array}{c} \text{Task critic: }v_{\text{task}}\left(s_{t}\right). \end{array} \end{array}$$

and it is trained by maximization of the expected reward predicted. Thanks to the world model, the reward is inferred in imagination, so the learning of the task actor-critic can happen fully in imagination, while the agent keeps exploring the environment (Sekar et al., 2020).

## 5 Experiments

We argue that the FOCUS object-centric world model and exploration strategy can be used to improve control in robotic manipulation, especially in sparse-reward settings. The experiments aim to empirically validate our argument by evaluating (i) the exploration performance of FOCUS compared to the state-of-the-art in world models and exploration, (ii) performance on sparse reward manipulation tasks, after an exploration stage. (iii) We validate the performance of the object-centric world model on dense reward tasks and present an additional analysis of the model, e.g. visualizing the reconstructions of the world model. Finally, we deploy FOCUS to a real-world setup.

### 5.1 Exploration-adaptation in sparse-reward tasks

**Figure 2:**
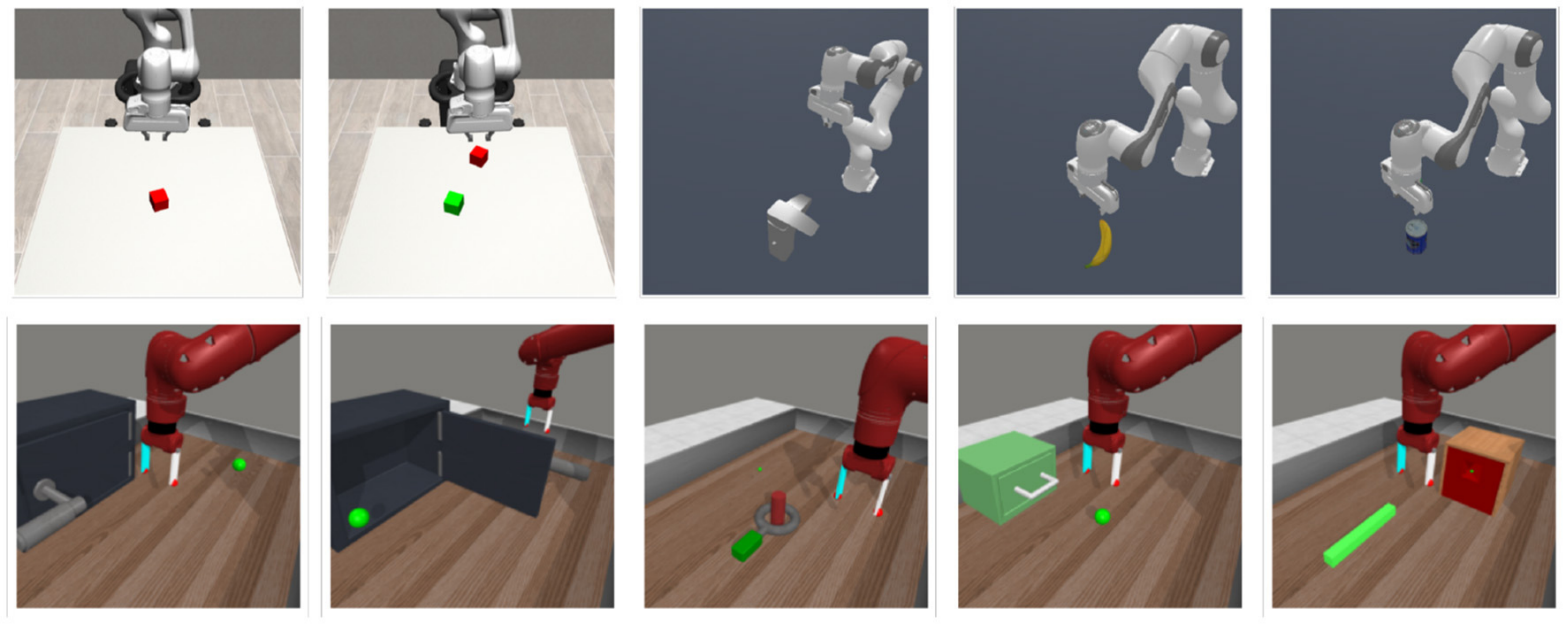
Manipulation environments. The 10 tasks we adopted are part of ManiSkill2 (MS), robosuite (RS) and Metaworld (MW). From **left–right**: Red cube (RS), RG cubes (RS), Faucet (MS), Banana (MS), Master Chef Can (MS), Door Open (MW), Door Close (MW), Disassemble (MW), Drawer Open (MW), Peg Insert (MW).

We adopt 10 tasks from three robotic manipulation benchmarks (shown in Figure 2): ManiSkill2 (Gu et al., 2023), robosuite (Zhu et al., 2020) and Metaworld (Yu et al., 2019). Both ManiSkill and robosuite provide segmentation masks as an (optional) input for the agent, while Metaworld does not. Thus, we adopted fastSAM (Zhao et al., 2023) to extract segmentation masks in those tasks, an evaluation setting that serves us the purpose of a test field for real-world experiments. The object of interest is prompted using text (Cheng et al., 2023), providing the name of the object in the scene. The masking produced by the SAM model is treated as the object masking, while the negative of it as background masking.

We compare FOCUS against three exploration strategies: Plan2Explore (P2E) (Sekar et al., 2020), Active Pre-training (APT) (Liu and Abbeel, 2021) and Random actions. For fairness with P2E and FOCUS, both APT and Random are implemented on top of a DreamerV2 world-model-based agent, following (Rajeswar et al., 2023) and using their open-source code. The hyperparameters are the same used for DreamerV2 (Hafner et al., 2021), with the exception of the batch size and sequence length, both equal to 32.

For the implementation of FOCUS, we introduced an object latent extractor unit consisting of a 3-layer MLP with a dimensionality of 512. The object-decoder network resembles the structure of the Dreamer's decoder, the depth factor for the CNN is set to 72. The K-NN filter adopted for the entropy approximation uses a K-nearest neighbors factor of K = 30.

#### 5.1.1 Exploration

To compare the performance of different exploration strategies for manipulation, we chose a set of metrics that are related to interactions with objects:

- *Contact (%):* average percentage of contact interactions between the gripper and the objects over an episode.
- *Positional displacement (m):* cumulative position displacement of all the objects over an entire episode.
- *Angular displacement (rad):* cumulative angular displacement of all the objects over an entire episode.

**Figure 3:**
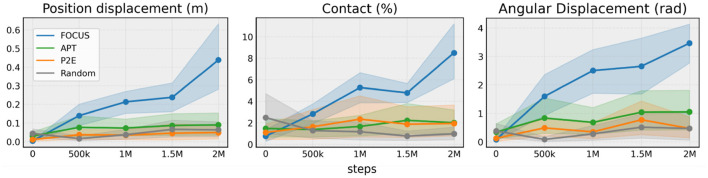
Exploration performance. Comparing exploration metrics across 10 tasks from ManiSkill2, robosuite and Metaworld. Experiments are run with three seeds per task and aggregated in a statistically sound way using RLiable (Agarwal et al., 2021).

In Figure 3, we observe that FOCUS interacts with objects much more assiduously than the other approaches, with the exploration performance consistently increasing over time. APT and P2E perform similarly and they only slightly perform better than Random, showing the importance of focussing on objects when exploring a robotic manipulation environment.

#### 5.1.2 Sparse reward tasks fine-tuning

**Figure 4:**
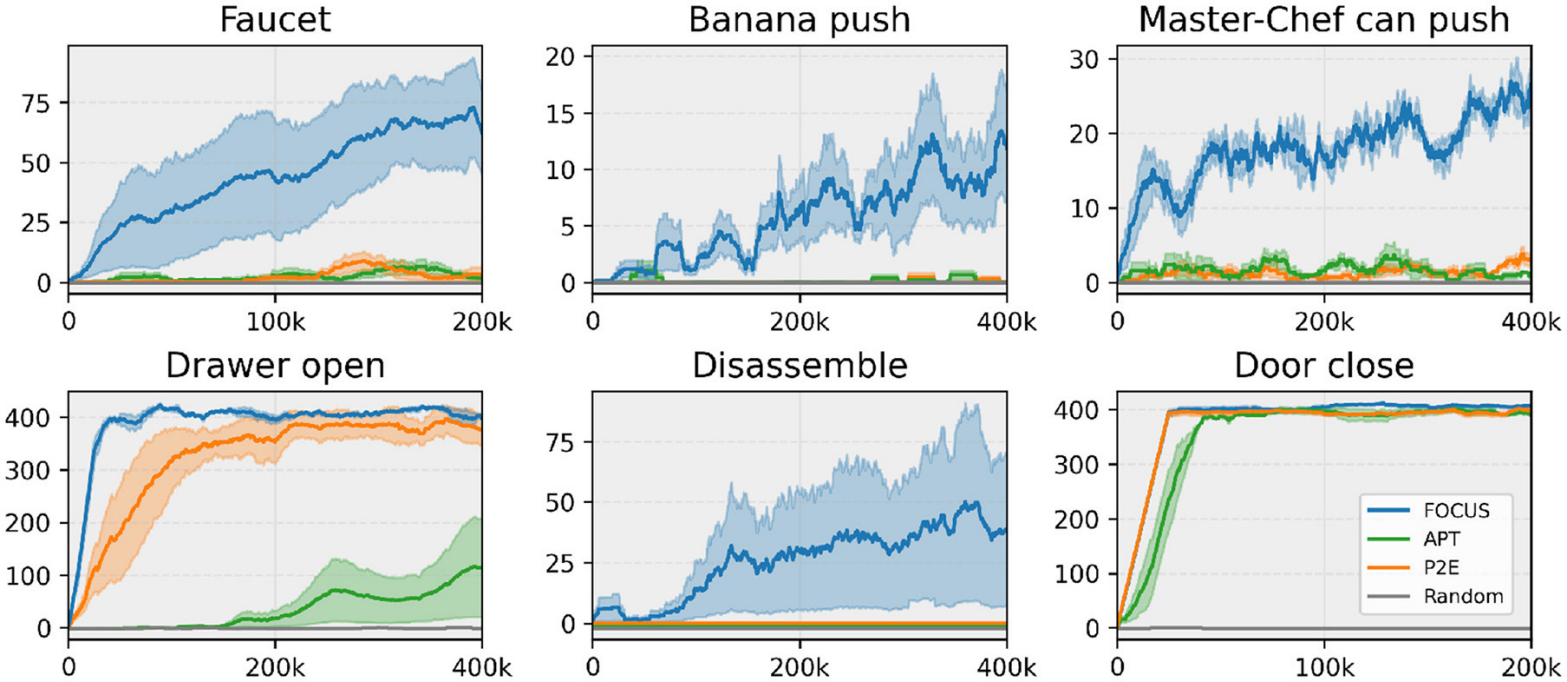
Sparse task fine-tuning performance. Comparing fine-tuning performance across tasks from ManiSkill2 (Faucet, Banana, Master Chef Can) and Metaworld (Drawer, Disassemble, Door). Experiments are run with three seeds.

During the exploration stage, all agents explore different actions in the environment, discovering the dynamics and reward function for a given task. However, the agents make no use of the task rewards during the exploration stage. After exploring the environment for 2M environment steps, we adapt the task actor-critic, using the rewards found during the exploration stage and allowing an additional (smaller) number of environment interactions for fine-tuning the agent and perfecting the task. The adaptation curves for six tasks, showing episode rewards over time, are presented in Figure 4.

The results show that FOCUS is the method that makes the most significant progress across all tasks. This proves that the agent consistently found sparse rewards in the environment, making adaptation to a given task easier. In support of this hypothesis, the fine-tuning performance starts increasing almost immediately in all tasks despite the sparse nature of the rewards. As for the other methods, we observe that Plan2Explore and APT were able to consistently find rewarding interactions only in a few tasks (Drawer Open, Door Close), where they perform well and similarly to FOCUS. Given a sparse reward signal, and not a dense one, it makes it hard for the methods with a limited exploration strategy to achieve good performance when deployed for fine-tuning. Instead, Random, being the most naive exploration strategy, barely found any rewards, making fine-tuning in sparse rewards settings difficult.

### 5.2 Additional analysis

We have developed object-centric world models to improve the way objects' information is represented in the world model, by leveraging a structured latent representation. We perform an additional analysis, to show that objects' prediction improves when employing object-centric structured world models, compared to using a “flat” latent structure, and to validate the information contained in the latent object states.

#### 5.2.1 Comparison to “flat” world models

**Figure 5:**
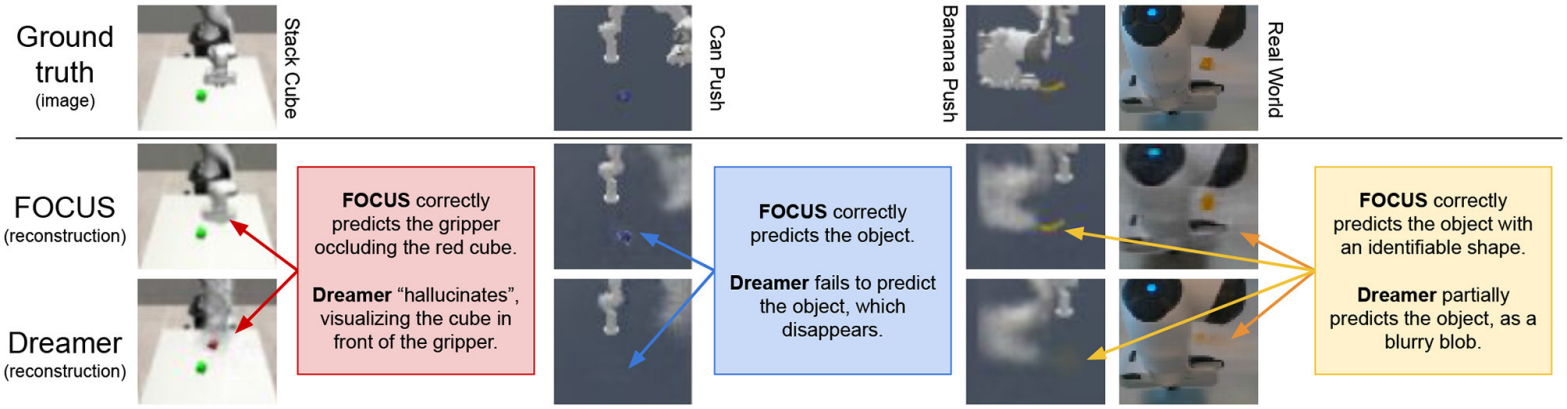
Visualizing reconstructions. Comparing reconstructions details of FOCUS's object-centric model and DreamerV2's world model on different environments (RG cube, Master Chef can, Banana, Real World cube). Images and reconstructions are provided with the same resolution as in the models, which is 64x64.

**Figure 6:**
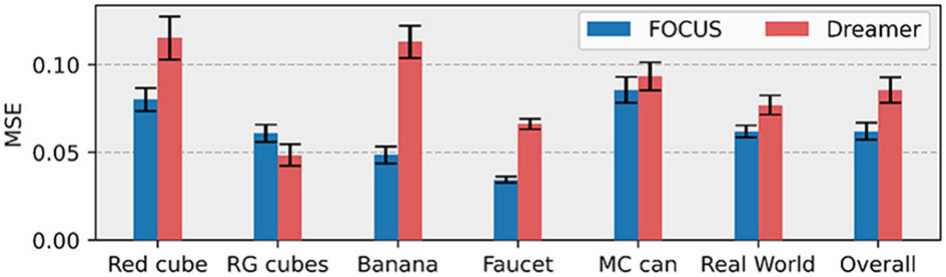
Object prediction errors. For each task, MSE is computed over the masked RGB image. Masking is obtained by dilatation (3 pixels) of the object's segmentation mask. Results are averaged over the 10 evaluation episodes.

Objects' size in the workspace is generally smaller than other elements, e.g. the robot, the table, and the background. When using a “flat” representation of the world, as Dreamer (Hafner et al., 2021) does, visual information about objects might be lost in the compression due to the encoding-decoding process of the world model. Qualitative reconstructions from the decoder of FOCUS are compared to reconstructions of Dreamer in Figure 5. Thanks to the explicit object's modeling FOCUS is able to reconstruct accurately any objects in the scene. Dreamer fails in many of these scenarios, especially in case of small objects, with poor visual contrast with respect to the background. In both Master Chef Can and Banana environments, Dreamer approximates each object in the scene with a cloudy presence, reflecting the lack of significant error signal to achieve a detailed reconstruction. To quantify the different performances in objects' predictions, we show the prediction error in the image area surrounding objects, in Figure 6. FOCUS is consistent in delivering more accurate object predictions.

**Figure 7:**
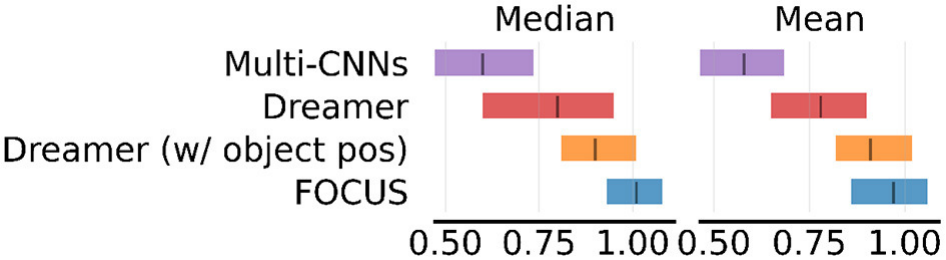
Dense reward performance. Results of dense reward experiments across 6 tasks. Experiments are run for 2M steps with three seeds per task and aggregated using RLiable (Agarwal et al., 2021).

*Do better object predictions yield better manipulation performance?* In order to isolate the problem of learning manipulation tasks from exploration, we compare FOCUS and Dreamer performance on a set of six dense-reward tasks: Drawer Open, Door Open, Door Close, Lift Cube, Stack Cube, and Turn Faucet. This comparison allows us to determine whether the improved object prediction performance is enough to generally improve performance in these tasks, independently of the exploration performance. We consider three baselines for these tasks: *Dreamer*, with the same set of observations provided to FOCUS, *Dreamer (w/ object pos)*, with additional object position information (x,y,z), and *Multi-CNNs* (Yoon et al., 2023) from the OCRL implementation (Yoon et al., 2023), as a model-free RL baseline using an object-centric representation. MultiCNNs extracts an object-centric representation from single observations (no temporal information), and it uses it to train a model-free PPO agent (Schulman et al., 2017). In Dreamer (w/ object pos), we concatenate object position to the proprioception of the agent. To account for the difference in dimensionality between this low-dimensional vector (proprioception + object position) and the large image matrix (64x64x3), we scale the proprioception loss term by a factor of 100. In Figure 7, we compare the final normalized performance in terms of episode rewards.

We observe that FOCUS obtains the highest median and mean performance. This supports the hypothesis that object-centric representations for world models generally improve RL performance for manipulation. When positional information is provided to Dreamer, this helps to improve performance since it is easier for the system to track objects' positions. Still, FOCUS shows an edge in performance, given the higher amount of implicit information available (e.g. orientation, contact, color, ...). Multi-CNNs struggle compared to the other approaches. We speculate this is linked to the lack of temporal consistency in the representation and to the adoption of a less efficient model-free learning strategy.

#### 5.2.2 Information partitioning

**Figure 8:**
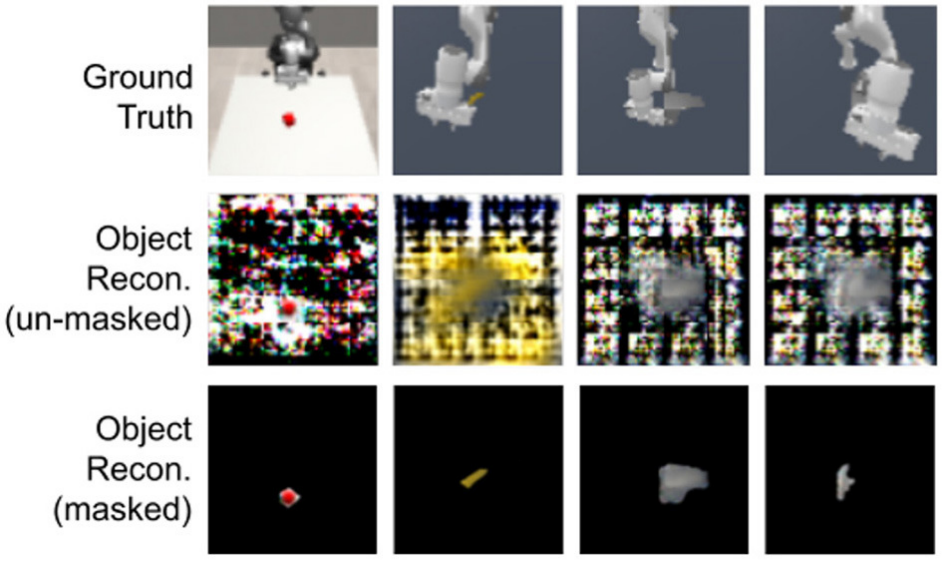
Object reconstructions. Unmasked and Masked objects reconstructions of FOCUS. Environments considered are Red cube, Banana, and Faucet. Images and reconstructions are provided with the same resolution as in the models, which is 64 × 64.

We assess whether FOCUS is correctly partitioning the information about each object into their respective latent while storing no additional information from the other elements in the scene. To get a glimpse into the information separation of FOCUS, we decode the information from the object's latent and we report some examples in Figure 8.

It is evident that the object latent is storing visual information about the object, capturing only a small amount of information from the rest of the image. The “leaked” information is present mostly in the area surrounding the object and we believe is due to the segmentation masks' quality. In the last two columns, we also show examples of occlusion behaviors (partial and full occlusion) by the robotic arm. Despite not seeing the object fully, FOCUS disentangles the object information from the robotic arm and can reconstruct the full unmasked object from the occluded views.

### 5.3 Real-world object-centric world model

We deploy FOCUS on a Franka Emika robot arm setup. The main issue in the real world comes from the absence of segmentation masks. Similarly to how we did for the MetaWorld experiments, we can adopt the fastSAM model (Kirillov et al., 2023; Zhao et al., 2023) to obtain segmentation masks, given a text prompt (Cheng et al., 2023).

**Figure 9:**
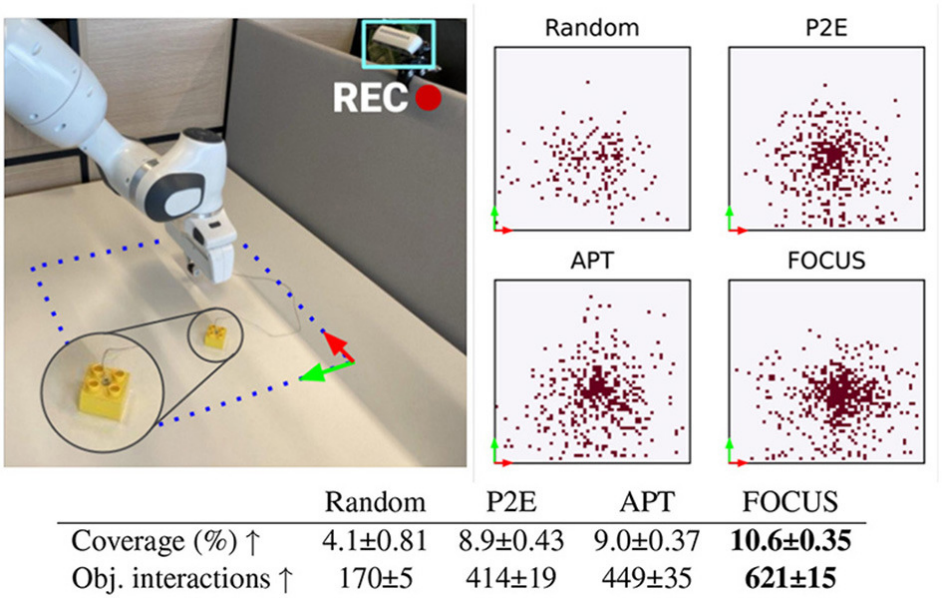
Real-world exploration. **Top-Left**: Real-world setup with delimited workspace. **Top-Right**: Positions reached by the cube during the finetuning phase for each method. **Bottom**: number of interactions between the manipulator and the cube, over 3 training seeds.

To evaluate the performance of the object-centric world model in a real-world setting, we designed a simple environment featuring a yellow brick placed on a tabletop, as shown in Figure 9. The cube, attached to the robot's end-effector by a string, serves as the primary interactive element. Each episode lasts for 100 steps, after which the robot resets to a designated position, bringing the cube back to approximately the center of the workspace. The robotic arm operates within a constrained 2D plane, indicated by the blue dotted line in Figure 9, with its end-effector height fixed above the tabletop. The robot's gripper remains closed and is not controllable, enabling it to interact with the cube exclusively through pushing movements. The restrictions imposed are for safety reasons due to the nature of exploration, but also to reduce the action space and therefore the amount of data collection required to model the environment.

In order to warm up the training of the world model, we pre-train all agents using a dataset of observations collected by using random actions (50 k interactions, approx 24 h of robot time). We use this dataset to pre-train each world model and exploration strategy for 500 k training steps (both the world model and the policy are updated at every step).

In Figure 6 (second to last histogram), we compare the object reconstruction error of Dreamer and FOCUS for the real-world scenario after pre-training. In general, the implicit segmentation knowledge makes it for more dynamically consistent reconstructions when compared to Dreamer. The latter can sporadically present artifacts (as depicted in the last column of Figure 5) in the reconstruction, especially for trajectories where there is interaction between the objects and the manipulator.

#### 5.3.1 Exploration evaluation

To evaluate the exploration capabilities of FOCUS in a real-world robotic setting, we fine-tune the pre-trained model for real-time exploration on a robotic arm. The finetuning process spans 10k steps, with each episode consisting of 100 steps. We compare FOCUS against the same exploration baselines proposed during the simulation experimentation, thus P2E, APT, and Random. Results are shown in Figure 9. To confirm what was seen during the simulation experiments, FOCUS has the highest interaction score with the object. The performance gap in terms of interaction between FOCUS and the other baselines is smaller compared to the simulated experiments due to the more simplistic setup adopted for the real-world scenario. The distribution of the object's position achieved during the fine-tuning phase is shown in the top-right part of Figure 9. FOCUS has the highest coverage of positions in the workspace, with the highest concentration around the center of the workspace.

## 6 Discussion

We presented FOCUS, an object-centric model-based agent that eagerly discovers interactions with objects, enabling one to learn manipulation tasks more efficiently. In our evaluation, we found that not only FOCUS enable solving more sparse reward tasks, but also that the object-centric representation generally improves objects' prediction and manipulation performance.

### 6.1 Limitations

In our exploration experiments we interact with the environment for 2M steps. All methods require first learning an adequate world model for the explorative agent to be able to robustly imagine which action is going to give the maximum explorative outcome. Indeed, FOCUS starts to show an edge over the other methods after 500 K explorative steps. This consistent amount of training steps makes it challenging to have a full deployment in a complex real-world environment. Nonetheless, exploration approaches can be applied in real-world setups, by simplifying the environment drastically, e.g. restricting the action space (Pathak et al., 2019) or employing high-level actions (Mazzaglia et al., 2024).

The primary limitation of FOCUS is its scalability when applied to scenes containing multiple objects of interest, e.g., more than 2. Since the model depends on segmentation masks to isolate the information for each object, each object reconstruction requires an additional output map, both for the segmentation weights and the RGB channels. This approach results in a larger computational and memory footprint that, despite providing higher performance, is less scalable. For future work, it would be interesting to investigate methods to isolate object information that use more compute-efficient representations, such as deep latent particles (Daniel and Tamar, 2022; Haramati et al., 2024), doing so would retain the benefits of the object-centric approach, while relaxing the computational requirements.

## Data Availability

Publicly available datasets were analyzed in this study. This data can be found here: https://github.com/StefanoFerraro/FOCUS.

## Footnotes

^1^The scene, with objects masked out, is also considered a “special object”. *N* is a chosen parameter, related to the objects of interest in the scene.
