# Supplementary material for: FOCUS: object-centric world models for robotic manipulation
Source: Front Neurorobot. 2025 Apr 30;19:1585386. doi: 10.3389/fnbot.2025.1585386 (PMC12075287; doi:10.3389/fnbot.2025.1585386)
Supplement: Supplementary file 1 [file Data_Sheet_1.pdf]

APPENDIX

Object-centric World Models training

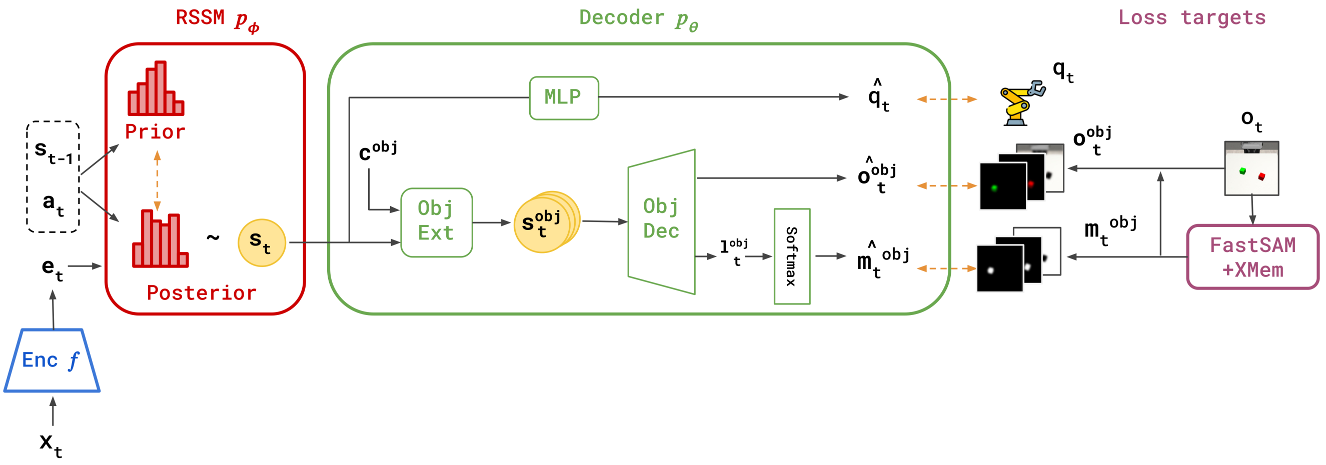

Figure 1. Object-centric World Models training. Detailed representation of the object-centric world models training.

In Figure 1, we detail the object-centric world model learned by focus, highlighting its training pipeline. Inputs x are encoded and transferred to the RSSM, which represents the latent state probability s as a categorical distribution. Latent states are sampled and fed to the decoder, which reconstructs the proprioceptive information of the robot, through an MLP, and extracts object-centric latents s^{obj}. These object-centric representations are decoded into object masks and RGB reconstructions. All reconstructions are compared to the original inputs. Prior latent's are compared to the posterior latent, to learn to reconstruct future states only by the history of latent states and actions.

Algorithm

Algorithm 1 FOCUS: Online training

Require: Initial agent modules: world model, exploration actor-critic, task actor-critic
Require: Initial model state s_0
Require: Initialized environment
if no replay buffer available **then**
    Initialize replay buffer.
end if

`// Pre-training`
for $t = 0, \dots, N_{PT}$ **do**
    Draw action from the exploration actor, $a_t \sim \pi_{expl}(a_t|s_t)$
    Apply action to the environment, $x_{t+1} \sim P(\cdot|s_t, a_t)$
    Add transition to replay buffer,
    if $t \mod \tau = 0$ **then**
        Update world model parameters ϕ, θ on the data from the replay buffer
        Update actor-critic parameters π_{expl}, v_{expl} in imagination, maximizing r^{obj}_{expl}
        Update actor-critic parameters π_{task}, v_{task} in imagination, maximizing r_{task}
    end if
end for
Output pre-trained modules $\{\pi^{PT}_{task}, v^{PT}_{task}, p^{PT}_{\theta}, p^{PT}_{\phi}\}$

`// Fine-tuning`
Load pre-trained modules $\{\pi^{PT}_{task}, v^{PT}_{task}, p^{PT}_{\theta}, p^{PT}_{\phi}\}$
Initialize replay buffer

for $t = 0, \dots, N_{FT}$ **do**
    Draw action from the task actor, $a_t \sim \pi_{task}(a_t|s_t)$
    Apply action to the environment, $x_{t+1} \sim P(\cdot|s_t, a_t)$
    Add transition to replay buffer,
    if $t \mod \tau = 0$ **then**
        Update world model parameters ϕ, θ on the data from the replay buffer
        Update actor-critic parameters π_{task}, v_{task} in imagination, maximizing r_{task}
    end if
end for
Output fine-tuned modules $\{\pi^{FT}_{task}, v^{FT}_{task}, p^{FT}_{\theta}, p^{FT}_{\phi}\}$
